# Size-selective separation and overall-amplification of cell-free fetal DNA fragments using PCR-based enrichment

**DOI:** 10.1038/srep40936

**Published:** 2017-01-19

**Authors:** Qiwei Yang, Zhenwu Du, Yang Song, Sujie Gao, Shan Yu, He Zhu, Ming Ren, Guizhen Zhang

**Affiliations:** 1 Research Center of Second Hospital, Jilin University, Changchun 130041, China; 2 Orthopedics Institute of Second Hospital, Jilin University, Changchun 130041, China; 3 Obstetrics and Gynecology of Second Hospital, Jilin University, Changchun 130041, China

## Abstract

This study aimed to establish a method for the selective amplification of cell-free fetal DNA (cffDNA) in maternal plasma and preserve the integrity of DNA fragments during amplification, thereby providing a sufficient amount of cffDNA to meet the requirement of routine non-invasive prenatal testing. We amplified DNA molecules in a one-reaction system without considering their particular sequences and lengths (overall amplification) by using PCR-based enrichment. We then modified PCR conditions to verify the effect of denaturation temperature on DNA amplification on various lengths of DNA (selective overall amplification). Finally, we used an optimum temperature range to amplify cffDNA selectively. Amplification results were validated by electrophoresis and real-time quantitative PCR. Our PCR-based enrichment efficiently amplified all DNA fragments with differing lengths within a single reaction system, as well as preserving the integrity of the DNA fragments. cffDNA was significantly amplified along with the selective amplification of small fragment maternal plasma DNA in an appropriate range of denaturation temperatures. We have established a PCR-based method for the simultaneous enrichment and amplification of cffDNA in order to meet the requirements of high cffDNA quantity for routine non-invasive prenatal testing.

The discovery of cell-free fetal DNA (cffDNA) in maternal plasma has greatly promoted the development of non-invasive prenatal diagnosis^1^. However, the concentration of cffDNA in maternal plasma is extremely low and accounts for only 2–19% of the total maternal plasma cell-free DNA^2^,^3^, and varies obviously among individuals. When the proportion of cffDNA in the maternal circulation is below 4%, even with next generation sequencing (NGS) technology, which has a high sensitivity, obtaining sufficient accuracy for current non-invasive prenatal testing (NIPT)^4^ is challenging. Furthermore, DNA sequencing or real-time quantitative polymerase chain reaction (qPCR) is often associated with low sensitivity. In addition, cffDNA is mixed with maternal derived cell-free DNA, and current studies on cffDNA are carried out under the background interference of large amounts of maternal derived cell-free DNA. These limitations restrict the application of cffDNA in clinical testing or diagnosis. Therefore, for wider clinical applications of cffDNA for accurate routine testing, it is advisable to eliminate the background interference of maternal derived cell-free DNA and increase the content and abundance of cffDNA.

cffDNA molecules are fragmented molecules and show a fragment size distribution of two peaks with roughly equal height at 143 and 166 bp. Generally, all cffDNA fragments are shorter than 300 bp and approximately 20% of maternal cfDNA fragments are longer than 300 bp^5^,^6^,^7^,^8^. It is therefore possible to enrich cffDNA by collecting maternal plasma cell-free DNA fragments with a length shorter than 300 bp. Electrophoresis has been used to separate short cffDNA fragments from large maternal derived cell-free DNA fragments, however precision is extremely low^9^,^10^. However, recent studies have addressed the issue of separating cffDNA from maternal cfDNA using alternative methods, such as microfluidics^11^ and silica particles^12^.

Amplified fragment length polymorphism (AFLP)^13^,^14^ is a PCR-based technique that can selectively amplify restriction fragments^15^,^16^,^17^. The basic procedure of AFLP is divided into three steps. First, genomic DNA is digested with one or more restriction enzymes. Second, all restriction fragments are ligated with restriction half-site specific linkers to the sticky ends. Finally, fragments are amplified with two PCR primers complementary to the linker and restriction site sequences. The denaturation temperature (Tm) of DNA molecule is related to a number of factors such as molecule length, base composition and ionic strength of the buffer^18^. In a single PCR system, the Tm of different DNA molecules is only related to their length and GC-content. If the impact of GC-content for PCR amplification can be reduced, the molecule length will become the major factor affecting denaturation temperature. Therefore, we suggest that if the denaturation temperature is reduced in an appropriate range, the amplification of larger DNA fragments will be suppressed without affecting the amplification of smaller DNA fragments; thereby achieving the purpose of amplifying smaller DNA fragments selectively.

The purpose of this study was to overcome the problems associated with existing DNA amplification technologies and establish a PCR-based enrichment protocol for the selective amplification of cffDNA by modifying amplification reaction conditions of AFLP. This established method is sufficient to provide a large amount of cffDNA to meet the requirement of routine testing.

## Materials and Methods

### Sample Collection and DNA Extraction

Peripheral blood (40 mL) was donated from 20 pregnant women (gestational age = 18.67 ± 0.58 weeks). Signed consent forms were obtained from each donor and all experiments were approved by the Ethical Committee of the Second Hospital, Jilin University, China (reference number: research examination No. 2014–026). All methods were performed in accordance with the relevant guidelines and regulations by including a statement in the methods section to this effect. Written informed consent was obtained. The blood samples were anticoagulated with EDTA. Plasma supernatant was separated from the entire blood by centrifugation at 1600 g for 10 min at room temperature. The supernatant was transferred into a new centrifuge tube to repeat centrifugation, followed by further centrifugation at 16000 g for 10 min to remove residual intact cells^9^,^19^. The supernatant was collected carefully for plasma cell-free DNA extraction by phenol-cholroform-isoamyl alcohol (240:24:1)^20^. The blood cells were collected for genomic DNA extraction by Blood DNA out Kit (Tiandz, China). The whole process was performed within 4 h of blood draw.

### Preparation of DNA fragments in different length

Primers were designed based on *Homo sapiens* chromosome 12, GRCh38 Primary Assembly (NC_000012.12) genomic DNA sequence. The primers were synthesized by Sangon Company (Shanghai, China) as shown in Table 1. Genomic DNA was amplified through these primers by Platinum HiProof Taq (HiGC & HiYield) (Cyagen, Shanghai, China) and GoTaq Colorless Master Mix (Promega, Madison, WI, USA) following the manufacturer’s manuals. All products were validated with 1% agarose gel electrophoresis. Target bands were purified by AxyPrep DNA Gel Extraction Kit (AXYGEN, Union City, CA, USA) following the manufacturer’s instruction to remove excess genomic DNA templates and primers in products. PCR products were treated with T4 Polynucleotide Kinase (Promega).

### Ligation of DNA fragments and double stranded unidirectional linkers

DNA fragments and linkers were ligated by T4 DNA Ligase (Promega) according to manufacturer’s instructions. The ligation products were validated with 20% PAGE electrophoresis. Image J software was used for analyzing the gray values of the bands.

### Overall amplification of DNA fragments in different length

Platinum HiProof Taq (HiGC & HiYield) was used for PCR according to manufacturer’s protocol. PCR was performed using the follow protocol: linker extension at 72 °C for 5 min, pre-denaturation at 94 °C for 3 min; 30 cycles of 94 °C for 20 sec, 72 °C (reduce 0.5 °C per cycle) for 20 s, 72 °C for 2 min; 30 cycles of 94 °C for 20 sec, 57 °C g for 20 sec, 72 °C for 2 min; finally, the reaction was extended at 72 °C for 5 min. The products were validated with 1% agarose gel electrophoresis.

### Overall amplification of maternal plasma total cell-free DNA

100 ng of cfDNA was blunt ended by T4 DNA Polymerase (Promega). Nuclease-free water was used as a negative control to replace cell-free DNA. Products were purified by AxyPrep PCRCleanup Kit (AXYGEN). dA Tailing Kit (Tiandz, China) was used for adding adenine bases to the 3′-ends. Nuclease-free water was used as negative control. The products were purified by AxyPrep DNA Gel Extraction Kit (AXYGEN), followed by ligation reaction. The ligated products were amplified by PCR with Platinum HiProof Taq (HiGC & HiYield), and checked by 1% agarose gel electrophoresis, followed by purification with AxyPrep PCRCleanup Kit. Quantitation of the PCR product was measured by NanoDrop 2000 Spectrophotometer (Thermo scientific). qPCR was performed by LightCycler 480 (Roche) using SYBR Premix Ex Taq (TaKaRa, Japan) to compare the relative levels of cfDNA.

### Effect of denaturation temperature on overall amplification of maternal plasma total cell-free DNA

PCR conditions, except pre-denature or denaturation temperatures, remained unchanged, as described earlier. Amplification products were validated with 1% agarose gel electrophoresis. qPCR was carried out to compare the relative quantity of varying length DNA fragments in overall amplification products.

**Figure 1:**
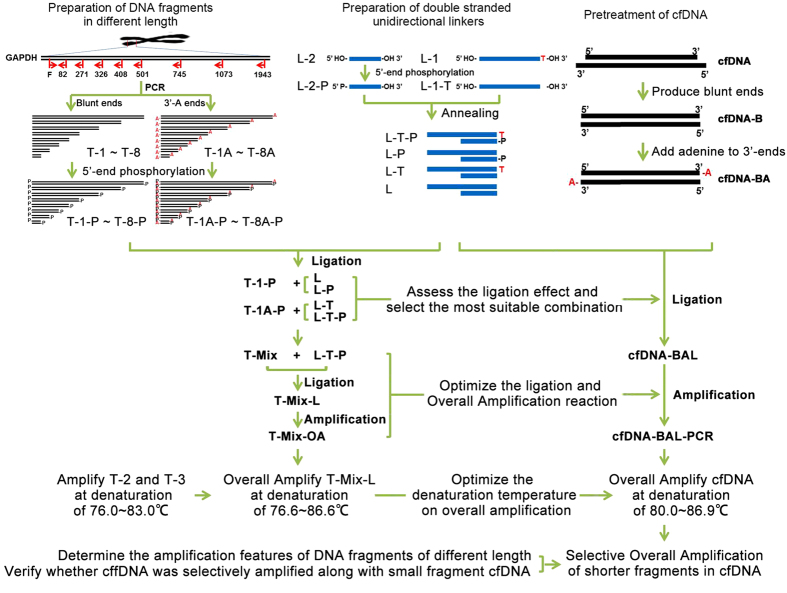
Brief flow chart of the present study. In “Preparation of DNA fragments in different length” section: DNA fragments in a series of length were obtained by PCR and phosphorylated at the 5′-ends. DNA fragments with blunt ends were marked as T-1~T-8 from small to large, respectively. DNA fragments with 3′-A overhanging ends were marked as T-1A~T-8A from small to large, respectively. In “Preparation of double stranded unidirectional linkers” section: four kinds of double stranded unidirectional linkers were made. L-T-P had 3′-T overhanging ends and 5′-end phosphorylation; L-P had blunt ends and 5′-end phosphorylation; L-T had 3′-T overhanging ends without 5′-end phosphorylation; L had blunt ends without 5′-end phosphorylation. In “Pretreatment of cfDNA” section: cfDNA was pre-treated by producing blunt ends and adding adenine to 3′-ends. Then, T-1-P was ligated with L or L-P while T-1A-P was ligated with L-T or L-T-P to assess the ligation effect and select the most suitable combination. Following this step, an equal amount of T-1A-P~T-8A-P were mixed (marked as T-Mix) and ligated with L-T-P (marked as T-Mix-L), followed by overall amplified (marked as T-Mix-OA) to optimize the ligation and overall amplification reaction. Afterwards, the optimized ligation reaction conditions were utilized for ligating pretreated cfDNA and double stranded unidirectional linkers (product marked as cfDNA-BAL), and the optimized amplification reaction conditions were utilized for overall amplifying cfDNA (product marked as cfDNA-BAL-PCR). T-Mix-OA was overall amplified at a series of denaturation temperatures to optimize the optimum temperature for selective overall amplification of shorter fragments in cfDNA. The amplification features of DNA fragments of different length were determined and cffDNA was verified in the selectively overall amplified small fragment cfDNA.

A brief flow chart summarizing the present study was shown in Fig. 1.

### Statistical analysis

SPSS 17.0 was used to perform statistical analyses on the Ct value. Ct values between two groups were compared using Student’s t-test. Multiple groups were compared using ANOVA. The two-sided p-value cutoff of 0.05 was used to decide whether there was statistical significance between values.

## Results

### Preparation of DNA fragments in different length and double stranded unidirectional linkers

**Figure 2:**
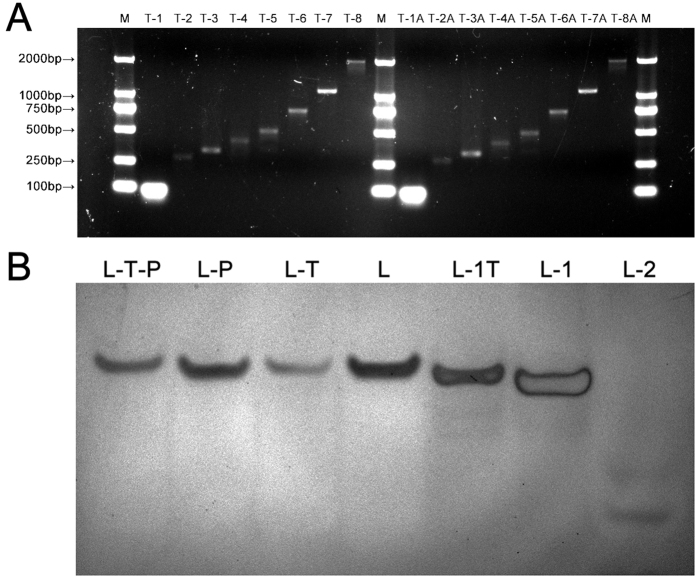
Preparation of DNA fragments with different length and double stranded unidirectional linkers. (**A**) Agarose electrophoresis image of DNA fragments with different length. M: DNA marker; T-1 ~T-8: DNA fragments with blunt ends; T-1A~T-8A: DNA fragments with 3′-A overhanging ends. (**B**) PAGE image of double stranded unidirectional linkers. L-T-P: the linker with 3′-T overhanging ends and 5′-end phosphorylation; L-P: the linker with blunt ends and 5′-end phosphorylation; L-T: the linker with 3′-T overhanging ends without 5′-end phosphorylation; L: the linker with blunt ends without 5′-end phosphorylation; L-1T, L-1 and L-2: oligonucleotides.

DNA fragments of different lengths were used to observe the ligation effect of substrate DNA and the improved double stranded unidirectional linker. We set one universal forward primer in the upstream region of genomic DNA and eight reverse primers in different locations downstream to obtain DNA fragments which were lengths of 82 bp, 271 bp, 326 bp, 408 bp, 501 bp, 745 bp, 1073 bp and 1943 bp thereby simulating cfDNA molecules in plasma. Our selection of this range of fragment lengths was mainly based on the fact that larger fragments have a relatively higher denaturation temperature range, and aimed to confirm our hypothesis that shorter fragments can be amplified selectively over longer ones at lower denaturation temperatures. Moreover, the reaction product can be clearly distinguished by agarose gel electrophoresis. In order to simulate cfDNA with blunt or 3′-A overhanging ends after different pretreatments. PCR was performed by using two types of DNA polymerases: Platinum HiProof Taq (HiGC & HiYield) and GoTaq Colorless Master Mix. Platinum HiProof Taq produces fragments with blunt ends (products marked as T-1~T-8); while the products of GoTaq Colorless Master Mix have 3′-A overhanging ends (products marked as T-1A~T-8A) (Fig. 2A). T4 Polynucleotide kinase was used to phosphorylate the 5′-ends of products to simulate the natural state of DNA molecules.

Corresponding to DNA fragments with blunt ends or 3′-A overhanging ends, we designed four variations of double stranded unidirectional linkers (Table 2) as described by Oberley *et al*.^21^ L-T-P (composed by L-1T and L-2-P) had 3′-T overhanging ends and 5′-end phosphorylation; L-P (composed by L-1 and L-2-P) had blunt ends and 5′-endphosphorylation; L-T (composed by L-1T and L2) had 3′-T overhanging ends without 5′-end phosphorylation; L (composed by L-1 and L-2) had blunt ends without 5′-end phosphorylation. Double stranded unidirectional linkers were annealed and checked by 20% PAGE electrophoresis. All the bands of double stranded unidirectional linkers and oligonucleotides were single and clear (Fig. 2B). Moreover, double stranded linkers (L-T-P, L-P, L-T and L) moved slower than single stranded oligonucleotides (L-1T, L-1 and L-2), indicating successful annealing of the double stranded unidirectional linkers.

### Ligation of DNA fragments and double stranded unidirectional linkers

**Figure 3:**
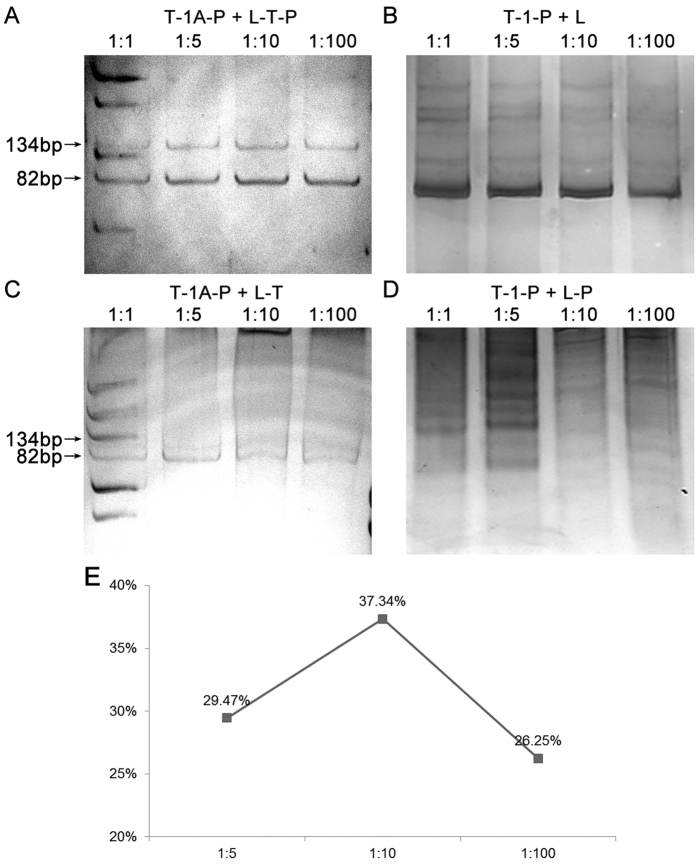
Ligation of DNA fragments and double stranded unidirectional linkers. (**A**) PAGE images of T-1A-P ligated with L-T-P. (**B**) PAGE images of T-1-P ligated with L. (**C**) PAGE images of T-1A-P ligated with L-T. (**D**) PAGE images of T-1-P ligated with L-P. (**E**) Ligation efficiency curve of T-1A-P ligated with L-T-P in different mass ratio. 1: 1, 1:5, 1:10 and 1:100: the mass ratio of DNA fragment: linker; 82 bp: the band of T-1A-P or T-1-P; 134 bp: the band of ligation products.

In order to assess the ligation effect and select the most suitable combination of substrate DNA pretreatment method and linker, the blunt ended DNA fragment (T-1-P) was ligated with blunt end linkers (L or L-P), while the 3′-A overhanging end DNA fragment (T-1A-P) was ligated with 3′-T overhanging end linkers (L-T or L-T-P), respectively. In order to investigate the optimum ratio of DNA fragments and linkers, the mass ratio of DNA fragments and linkers in the ligation reactions were set as 1:1, 1:5, 1:10 and 1:100. A single and specific ligation product (134 bp) was obtained in the reaction of T-1A-P with L-T-P with the ratio of 1:5, 1:10 and 1:100 (Fig. 3A), but the additional bands represented in the 1:1 fragment to linker reaction and no specific products were obtained in the other reactions (Fig. 3B–D). These additional bands may be caused by the unspecific random ligation between the fragments or linkers. These results indicate that the highest ligation specificity occurs when substrate DNA possesses 3′-A overhanging ends and the linkers have 3′-T overhanging ends with a phosphorylated 5′ end. The reactions without unspecific ligation products were chosen for investigating ligation efficiency. The ligation efficiency was calculated using the following formula: Ligation efficiency = gray values of 134 bp/(gray values of 134 bp + gray values of 82 bp) × 100%. The optimal mass ratio of DNA fragment to linker of 1:10 demonstrated the highest ligation efficiency (Fig. 3E).

### Overall amplification of DNA fragments in different length and maternal plasma total cell-free DNA

**Figure 4:**
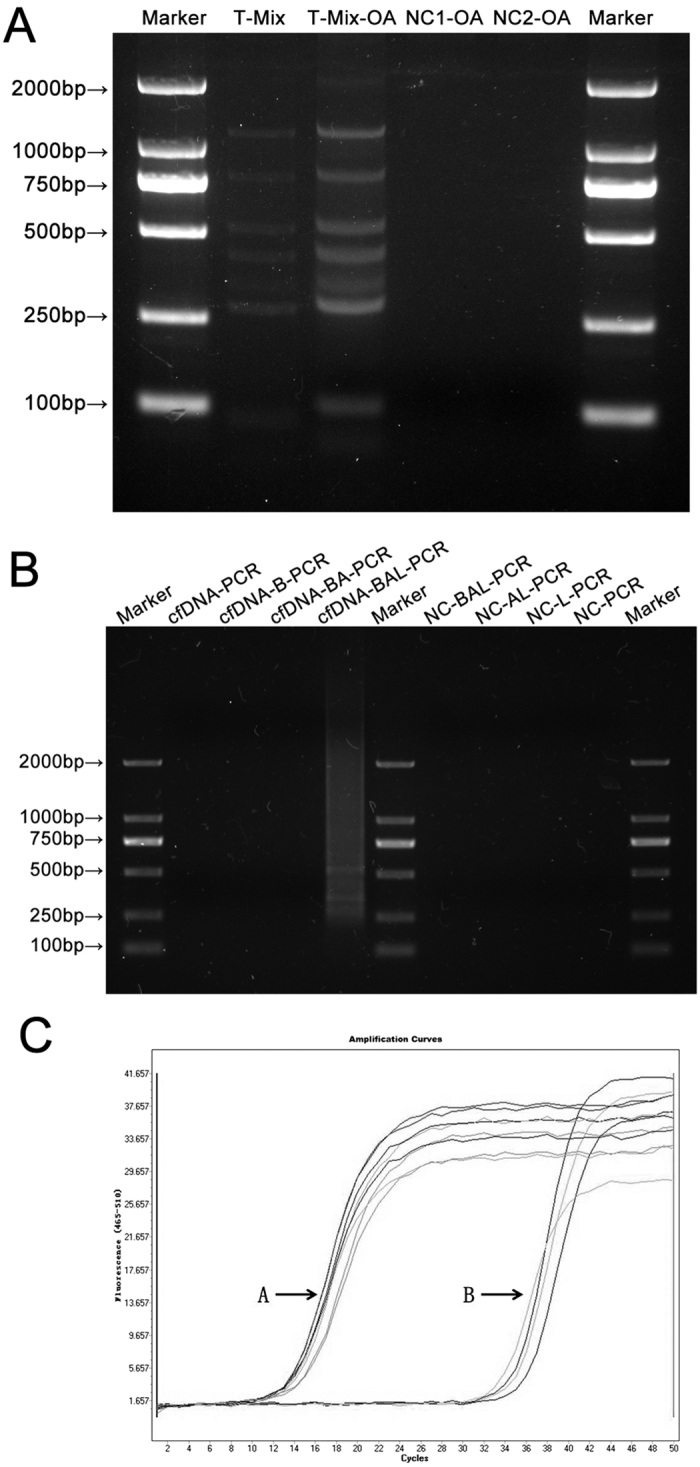
Overall amplification of DNA fragments of different length and maternal plasma total cell-free DNA. (**A**) Agarose electrophoresis image of DNA fragments with different length after overall amplification. Marker: DNA marker; T-Mix: the mixture of T-1A-P~T-8A-P; T-Mix-OA: the overall amplification product of T-Mix; NC1-OA: the overall amplification product of negative control 1; NC2-OA: the overall amplification product of negative control 2. (**B**) Agarose electrophoresis image of cfDNA after overall amplification. cfDNA-BAL-PCR: the overall amplification product of cfDNA; cfDNA-PCR, cfDNA-B-PCR and cfDNA-BA-PCR: the PCR products of pretreatment product in each stage; NC-BAL-PCR, NC-AL-PCR, NC-L-PCR and NC-PCR: the PCR products of negative control for pretreatment in each stage. (**C**) Amplification curves of cfDNA with or without overall amplification. (**A**) Amplification curves of cfDNA-BAL-PCR; (**B**) Amplification curves of cfDNA.

In order to optimize the ligation and overall amplification reaction, an equal amount of T-1A-P~T-8A-P (T-Mix) were mixed and ligated with L-T-P. The ligation products were marked as T-Mix-L. Nuclease-free water was used as negative controls to replace T-Mix (NC1) and replace L-T-P (NC2), respectively. As every DNA molecule that had been successfully ligated with linkers had the same sequence as L-1 in their 5′-ends, L-1 was used as a universal primer to amplify these DNA molecules in a single reaction system. To further examine this hypothesis, we amplified T-Mix-L using L-1 as a primer (product marked as T-Mix-OA) with Touchdown-PCR to reduce non-specific amplification. As shown in Fig. 4A, the band position of T-Mix-OA corresponded to the unamplified T-Mix, indicating that DNA fragments were efficiently and specifically amplified after ligation with linkers without considering their particular sequences and lengths. We therefore named this amplification method as “overall amplification”.

The above results demonstrated that our approach is able to amplify all DNA fragments of varying lengths within a single reaction system and the integrity of DNA fragments are protected to the maximum extent. Accordingly, we performed the overall amplification on cell-free DNA using this approach. Because cell-free DNA molecules in plasma originate primarily from apoptotic processes^5^ with overhanging ends, pretreatments were performed prior to ligation. T4 DNA Polymerase was used to produce blunt ends (marked as cfDNA-B). Subsequently, adenine bases were added to the 3′-ends of cfDNA-B molecules (marked as cfDNA-BA). cfDNA-BA was then ligated with L-T-P (marked as cfDNA-BAL), followed by PCR amplification using primer L-1 (marked as cfDNA-BAL-PCR). The results showed that cfDNA-BAL-PCR presented as a clear, continuous, symmetrical smear band (Fig. 4B), indicating successful overall amplification of cfDNA through ligation with linkers. Moreover, the content of two cfDNA samples and two overall amplification products from each cfDNA samples (cfDNA-BAL-PCR) were compared by qPCR through GAPDH sequence (Table 3). The amplification curves of cfDNA and cfDNA-BAL-PCR showed that the content of cfDNA was increased significantly following overall amplification (Fig. 4C).

### Effect of denaturation temperature on overall amplification

**Figure 5:**
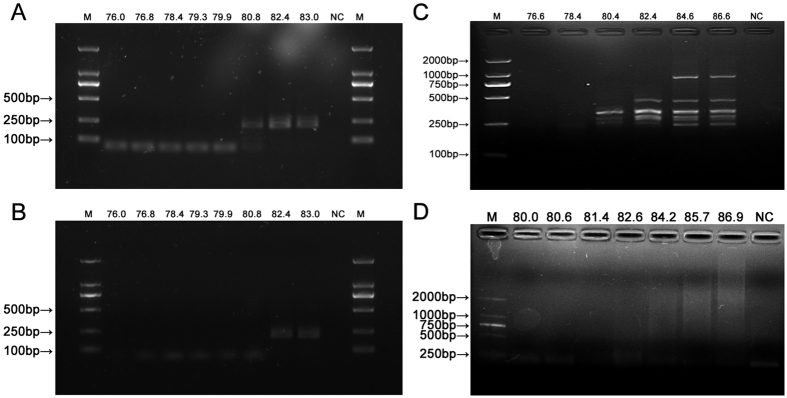
Effect of denaturation temperature on overall amplification. (**A**) Agarose electrophoresis image of PCR product using T-2 as templates in different denaturation temperatures. (**B**) Agarose electrophoresis image of PCR product using T-3 as templates in different denaturation temperatures. (**C**) Agarose electrophoresis image of overall amplification product of T-Mix-L in different denaturation temperatures. (**D**) Agarose electrophoresis image of overall amplification product of cfDNA in different denaturation temperatures. M: DNA Marker DL 2000; 76.0~86.9: Denaturation temperature (°C); NC: Negative control.

In order to optimize the denaturation temperature on overall amplification, two DNA fragments, possessing the same sequence, were used as templates to amplify the common sequence through a series of denaturation temperatures. To achieve this purpose, T-2 and T-3 were used as templates and F and R2 were used as primers (Table 1) for PCR at different denaturation temperature. Temperatures of 76.0 to 83.0 °C were used as pre-denaturation or denaturation temperatures and other conditions remained unchanged. When the denaturation temperature was between 76.0~79.9 °C, there were no expected bands in both T-2 or T-3 groups except for primer dimers. At the denaturation temperature of 80.8 °C, a band appeared with T-2 with some primer dimers, but T-3 could not be amplified. When the denaturation temperature was increased to the range of 82.4–83.0 °C, both T-2 and T-3 group were amplified and showed the expected bands (Fig. 5A and B). These results suggest that reducing the denaturation temperature of the PCR within a certain range can impede the amplification of large DNA fragments without affecting the amplification of small DNA fragments, and the temperature which impedes the amplification of DNA fragments larger than 300 bp may be within the range of 79.9–82.4 °C.

Next, the overall amplification of DNA fragments of different lengths was carried out with different denaturation temperatures. T-Mix-L was amplified using L-1 as the primer with a pre-denature or denaturation temperature of 76.6–86.6 °C, with the other conditions remaining unchanged. There were no bands when the denaturation temperature was in the range of 76.6–78.4 °C. When the denaturation temperature was increased to 80.4 °C, small fragments were amplified. When the denaturation temperature continued to increase, larger DNA fragments were also amplified (Fig. 5C). Finally, we attempted to amplify overall cfDNA through this approach in a theoretical denaturation temperature range and similar results were obtained (Fig. 5D). cfDNA-BAL was amplified using L-1 as primer with pre-denaturation or denaturation temperatures of 80.0–86.9 °C and other conditions remaining unchanged. There were no amplification products when the denaturation temperature was in the range of 80.0–81.4 °C. When the denaturation temperature was increased to 82.6 °C, small fragments shorter than 500 bp were amplified. When the denaturation temperature was increased to above 84.2 °C, larger fragment bands appeared gradually with increasing denaturation temperatures.

### Selective overall amplification of maternal plasma total cell-free DNA

These results confirmed our conjecture that if the denaturation temperature is reduced in an appropriate range, the amplification of larger DNA fragments will be suppressed without affecting the amplification of smaller DNA fragments, thereby achieving the purpose of selective amplification of smaller DNA fragments. We named this amplification method “selective overall amplification”.

**Figure 6:**
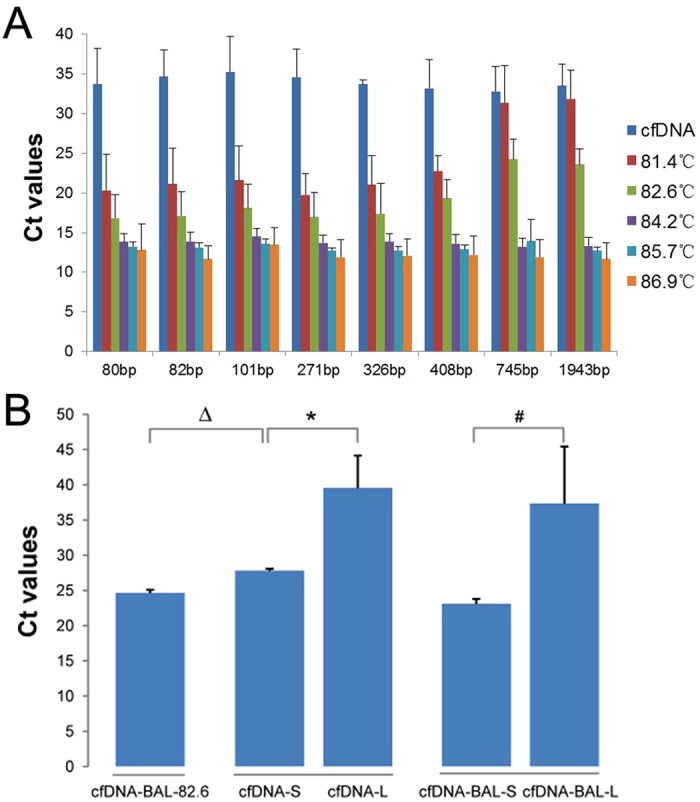
Determination of the relative quantity of DNA fragments of different length in overall amplification products by qPCR. (**A**) The amplification effect of different length fragments in the overall amplification products under different denaturation temperatures. 80 bp~1943 bp: The amplification products length were 80 bp~1943 bp; cfDNA: The cfDNA without overall amplification; 81.4 °C–86.9 °C: The templates were the overall amplification products with the denaturation temperature of 81.4 °C–86.9 °C. (**B**) The amplification profile of SRY gene (n = 6). ^Δ^Significant difference between cfDNA-BAL-82.6 and cfDNA-S (P < 0.05); *significant difference between cfDNA-S and cfDNA-L (P < 0.05): ^#^significant difference between cfDNA-BAL-S and cfDNA-BAL-L (P < 0.05).

In order to further determine the amplification features of DNA fragments of different length that can be overall amplified through different denaturation temperatures, qPCR was used to compare the relative quantity of DNA fragments of different lengths in overall amplification products. qPCR was performed with the denaturation temperatures of 81.4 °C, 82.6 °C, 84.2 °C, 85.7 °C, 86.9 °C and cfDNA as a template. The primers with product lengths of 80 bp, 82 bp, 101 bp, 271 bp, 326 bp, 408 bp, 745 bp and 1934 bp (Tables 1 and 3) were used as primers. qPCR results of cfDNA and PCR products at different denaturation temperatures are presented in Fig. 6A. The Ct values of cfDNA without overall amplification were the highest in each group, indicating cfDNA contains the minimum amount of DNA molecules. In 81.4 °C and 82.6 °C groups, the Ct values increased with the length of target fragments, reflecting that the content of larger DNA fragments (745–1943 bp) was lower than smaller fragments DNA (80–408 bp). In the 84.2 °C, 85.7 °C and 82.6 °C groups, the Ct values of each target fragments were similar, which indicated that the content did not differ among variable length DNA fragments in these groups. The melt curves were analyzed and unspecific amplification was not observed.

In order to verify whether cffDNA was selectively amplified along with small fragment cfDNA, we compared the relative quantity of sex-determining region Y (SRY) gene, which only exists in cffDNA derived from male fetuses. Six patients were detected to conceive a male fetus and included in the following study. cfDNA and the amplified product of cfDNA-BAL with the denaturation temperature of 86.9 °C were divided into two parts by 1% agarose gel electrophoresis at the position of 300 bps and extracted by AxyPrep DNA Gel Extraction Kit. The longer fragment sections were marked as “cfDNA-L” and “cfDNA-BAL-L”, the shorter fragment sections were marked as “cfDNA-S” and “cfDNA-BAL-S”. The amplification product of cfDNA-BAL with the denaturation temperature of 82.6 °C was purified by AxyPrep PCRCleanup Kit and marked as “cfDNA-BAL-82.6”. qPCR was used to detect the relative content of SRY gene (Table 3) in cfDNA-BAL-82, cfDNA-L, cfDNA-BAL-L, cfDNA-S and cfDNA-BAL-S. Figure 6B shows the Ct values of the SRY gene in cfDNA-L, cfDNA-S, cfDNA-BAL-L, cfDNA-BAL-S and cfDNA-BAL-82.6. The results demonstrated that the content of the SRY gene in fragments shorter than 300 bp (cfDNA-S, cfDNA-BAL-S and cfDNA-BAL-82.6) was significantly higher than in fragments longer than 300 bp (cfDNA-L and cfDNA-BAL-L). Furthermore, the content of SRY in cfDNA-BAL-82.6 was significantly higher than in cfDNA-S. These results suggest that in the process of selective overall amplification, short fragments of cfDNA and cffDNA were efficiently amplified.

## Discussion

In this study, in order to provide sufficient amounts of cffDNA to meet the requirement of routine testing, we improved the methodology of conventional AFLP. Restriction enzyme digestion was replaced by T4 DNA polymerase that converts the overhanging ends to blunt ends in order to pretreat substrate cfDNA. Subsequently, a dA Tailing Kit was applied to create 3′-A sticky ends at the end of cfDNA fragments. For the double stranded unidirectional linkers, we introduced a T base to create 3′-T ends at the connection end of the double stranded unidirectional linker thereby increasing the ligation specificity between linkers and substrate DNA and reducing self-ligation of substrate DNA molecules. Concurrently, we phosphorylated the 5′ end of connection end of substrate DNA. With these strategies, stable phosphodiester bonds at both ends of the double stranded DNA molecule were formed and the ligation efficiency between linkers and substrate DNA, as well as the stability of the ligation products were improved. In addition, during the amplification process, we utilized a form of DNA polymerase that has no 5′ → 3′ exonuclease activity and modified the reaction buffer thereby reducing the impact of GC-content on PCR amplification. Our results suggest that the fetal fraction would be increased in light of maternal cfDNA fragments longer than 300 bp not being amplified. Furthermore, the integrity of DNA fragments during the amplification was preserved. Our established method may lay a foundation for the use of cffDNA in NIPT in a cost effective manner.

There are currently several methods to separate DNA fragments with varying sizes. Gel electrophoresis is a commonly used method, however, it is limited by a time-consuming process, low recovery efficiency (<60%) and susceptibility to contamination. Hahn *et al*.^11^ described an electrokinetic trapping-based microdevice which can be used for enriching and pre-concentrating DNA from a mixture of nucleic acids of different sizes and preparative separation of the concentrated DNA by size. This microdevice provides an integrated, inexpensive, and easy-to-use solution by which fetal DNA is concentrated 4–8 times compared to pre-concentration samples. This method can improve the relative quantity of cffDNA, but cannot increase absolute quantity. A recent study showed that Lys-silica particles can also be utilized for size-selective separation of DNA mixtures by adjusting the electrostatic interactions between DNA fragments and the lysine-coated surface of the silica particles alongside pH especially ionic strength^12^. However, plasma contains a plethora of constituents including proteins and ions, and is significantly different between patients. Therefore, it is important to evaluate its application in plasma. Furthermore, at the optimum environmental pH and ionic strength, Lys-silica particles preferentially adsorb long DNA fragments. When Lys-silica is used for selective separation of short DNA fragments, which are mainly cffDNAs, adjustment of environmental pH and ionic strength is inevitable. Thereafter, alteration of pH and ionic strength may result in loss or damage of DNA structure. To the best of our knowledge, there is no efficient and specific technique to separate and enrich cffDNA from maternal plasma for NIPT in a single process. Our approach improved enrichment and amplification of cffDNA, as well as reduced the background level of maternal cfDNA. This process overcame the limitation of fetal specific gene markers. Our approach can produce large amounts of cffDNA in a simple process in order to meet the requirement of routine clinic NIPT, especially qPCR-based methods.

Our method has several potential advantages in qPCR based NIPT, such as reduced detection costs, lower requirements for the technology and equipment. Moreover, it may increase the sensitivity of aneuploidy disease detection. Thus it is affordable to the majority of primary hospitals. However, multiple steps, tedious processes, and time-consuming methods are some of the disadvantages and limitations associated with this technique. As multiple purification steps are required, cfDNA loss is unavoidable. Loss of cfDNA through purification steps affects NGS based methods where PCR duplicates are removed during bioinformatics analysis prior to copy number variation (CNV) analysis. Although our method may not improve NGS based methods, which are based on CNV analysis, it may greatly improve the sensitivity and specificity of qPCR based NIPT methods. More importantly, our observation that shorter fragments can be amplified selectively over longer fragments at lower denaturation temperatures may open an interesting new research direction. In our future studies, we will improve the accuracy of selective amplification, with an aim to accurately distinguish fragments with smaller length differences and to investigate the relationship between length and base composition of DNA fragment. Moreover, we will improve our methods to increase the efficiency and fidelity of selective amplification and the proportion of cffDNA in the overall amplification product. Furthermore, additional specific fetal fraction quantification methods will be used to determine an effective increase in fetal fraction. We will investigate the proportion of cffDNA in the amplification product via paternal-specific inherited molecular markers by dPCR or MPS methods. In addition, we will further validate this method using current standard NIPT testing on samples from pregnant women, carrying offspring with and without trisomy 21, 18 and 13.

## Conclusion

In summary, we successfully established a PCR-based enrichment method for selectively amplifying shorter fragments in a sample containing a mixture of DNA with differing fragment sizes, such as cfDNA, while preserving the integrity of DNA fragments during amplification. This method could be implemented for simultaneous enrichment and amplification of cffDNA, which may provide a large amount of cffDNA and remove long maternal cfDNA molecules in maternal plasma to meet the requirement of routine testing, especially qPCR based methods, and allow for the use of cffDNA in NIPT in a cost effective manner.

## Additional Information

**How to cite this article:** Yang, Q. *et al*. Size-selective separation and overall-amplification of cell-free fetal DNA fragments using PCR-based enrichment. *Sci. Rep.* **7**, 40936; doi: 10.1038/srep40936 (2017).

**Publisher's note:** Springer Nature remains neutral with regard to jurisdictional claims in published maps and institutional affiliations.

## Figures and Tables

**Table 1:** Primer information for preparing DNA fragments with different length.

Primer symbol	Primer sequence (5′-3′)	Product length (bp)	Symbol of product with blunt ends	Symbol of product with 3′-A overhanging ends
F	GAAGGTCGGAGTCAACGG	—	—	—
R1	AGGCTCGTAGACGCGGTT	82	T-1	T-1A
R2	AGTAGGGACCTCCTGTTTCTG	271	T-2	T-2A
R3	CGAAAGGAAAGAAAGCGTC	326	T-3	T-3A
R4	GCTGCGCACTAGCATCCC	408	T-4	T-4A
R5	AGGCTTTCCTAACGGCTG	501	T-5	T-5A
R6	TGTCCTTTTCCAACTACCCA	745	T-6	T-6A
R7	GAAATCAGGAGTGGGAGCA	1073	T-7	T-7A
R8	GGAAGATGGTGATGGGATTT	1943	T-8	T-8A

Note: F: Forward primer; R1-R8: Reverse primers.

**Table 2:** Sequences of oligonucleotides.

Symbol	Sequence (5′-3′)
L-1T	GCGGTGACCCGGGAGATCTGAATTCT
L-1	GCGGTGACCCGGGAGATCTGAATTC
L-2	GAATTCAGATC
L-2-P	P-GAATTCAGATC*

^*^Phosphorylated modification on 5′ end.

**Table 3:** Primers for qPCR.

Primer symbol	Primer sequence (5′-3′)	Product length (bp)
GAPDH-F	GGACTGAGGCTCCCACCTTT	157
GAPDH-R	GCATGGACTGTGGTCTGCAA	
80-F	TGAAACATACGTTCCCAAAGAGTTT	80
80-R	CTCTCCTTCTCAGAAAGTGTGCATAT	
101-F	GTGCACCTGACTCCTGAGGAGA	101
101-R	CCTTGATACCAACCTGCCCAG	
SRY-F	AAAGGCAACGTCCAGGATAGAG	137
SRY-R	CCACTGGTATCCCAGCTGCT	
